# Narrowing the field: cancer-specific promoters for mitochondrially-targeted p53-BH3 fusion gene therapy in ovarian cancer

**DOI:** 10.1186/s13048-019-0514-4

**Published:** 2019-04-30

**Authors:** Katherine Redd Bowman, Ji Hoon Kim, Carol S. Lim

**Affiliations:** 1 University of Utah, 30 S 2000 E Room #301, Salt Lake City, UT 84112 USA; 2 New York University, 31 Washington Pl, New York, NY 10003 USA

**Keywords:** Ovarian cancer, p53, Gene therapy, Cancer-specific promoters, Mitochondrial targeting, hTERT, Ran, Brms1

## Abstract

**Background:**

Despite years of research, the treatment options and mortality rate for ovarian cancer remain relatively stagnant. Resistance to chemotherapy and high heterogeneity in mutations contribute to ovarian cancer’s lethality, including many mutations in tumor suppressor p53. Though wild type p53 gene therapy clinical trials failed in ovarian cancer, mitochondrially-targeted p53 fusion constructs, including a fusion with pro-apoptotic protein Bad, have shown much higher apoptotic potential than wild type p53 in vitro. Due to the inherent toxicities of mitochondrial apoptosis, cancer-specificity for the p53 fusion constructs must be developed. Cancer-specific promoters such as hTERT, hTC, Brms1, and Ran have shown promise in ovarian cancer.

**Results:**

Of five different lengths of hTERT promoter, the − 279/+ 5 length relative to the transcription start site showed the highest activity across a panel of ovarian cancer cells. In addition to − 279/+ 5, promoters hTC (an hTERT/CMV promoter hybrid), Brms1, and Ran were tested as drivers of mitochondrially-targeted p53-Bad and p53-Bad* fusion gene therapy constructs. p53-Bad* displayed cancer-specific killing in all ovarian cancer cell lines when driven by hTC, − 279/+ 5, or Brms1.

**Conclusions:**

Cancer-specific promoters hTC, − 279/+ 5, and Brms1 all display promise in driving p53-Bad* gene therapy for treatment of ovarian cancer and should be moved forward into in vivo studies. -279/+ 5 displays lower expression levels in fewer cells, but greater cancer specificity, rendering it most useful for gene therapeutics with high toxicity to normal cells. hTC and Brms1 show higher transfection and expression levels with some cancer specificity, making them ideal for lowering toxicity in order to increase dose without as much of a reduction in the number of cancer cells expressing the gene construct. Having a variety of promoters available means that patient genetic testing can aid in choosing a promoter, thereby increasing cancer-specificity and giving patients with ovarian cancer a greater chance at survival.

## Background

Despite initial response to surgery and chemotherapy, high recurrence rates [1, 2] and difficulty in screening mean that ovarian cancer has a 5-year survival rate of only 47.4% [3]. The most aggressive ovarian cancer subtype, high-grade serous carcinoma (HGSC), is also classified as the most lethal gynecological malignancy [4, 5]. Much of ovarian cancer’s lethality can be attributed to its high level of heterogeneity—over 15 different oncogenes can be mutated [6, 7], complicating targeted therapy—and ability to quickly develop resistance against chemotherapeutic agents. HGSC in particular often possesses resistance mutations such as increased ability to repair DNA damage and upregulated drug efflux pumps, as revealed by whole-genome sequencing of HGSC patients [6–8]. In particular, greater than 96% of HGSCs contain mutated p53—a key tumor suppressor protein—making loss of p53 function a key driver for HGSC development [6–8].

**Fig. 1:**
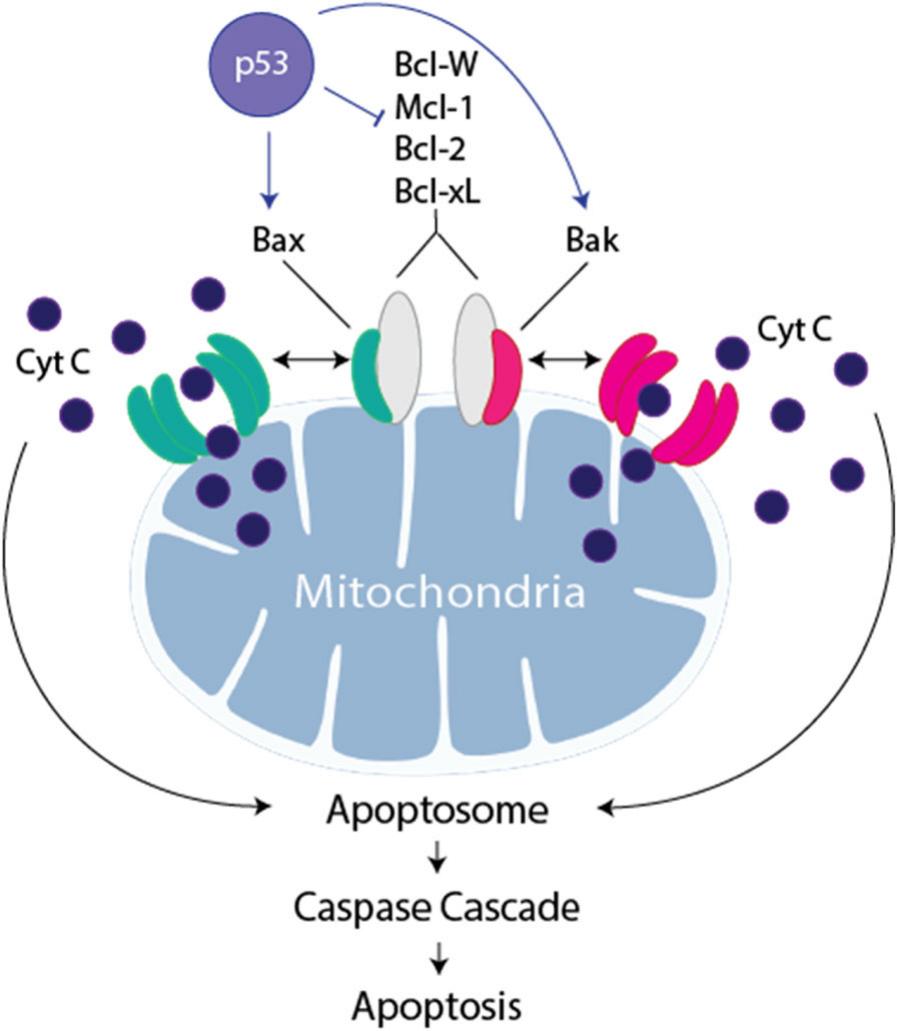
p53 at the Mitochondria. p53 activates pro-apoptotic factors Bak and Bax, which homo-oligomerize and recruit factors to form mitochondrial pores, leading to the release of cytochrome C, activation of the apoptosome, the caspase cascade, and apoptosis [10]. p53 also inactivates anti-apoptotic factors Bcl-W, Mcl-1, Bcl-2, and Bcl-xL, which function by binding Bak and Bax, thus preventing their homo-oligomerization and, therefore, apoptosis [38]

p53’s anti-tumor effect is well-known. Naturally, p53 localizes mainly to the nucleus, where it forms a tetramer and regulates apoptosis through the extrinsic (nuclear gene activation) pathway [9]. Additionally, a small amount of monomeric p53 localizes to the mitochondria, where it can rapidly induce apoptosis through the intrinsic pathway (Fig. 1) [10, 11]. Because a tetramer is necessary for nuclear activation, therapeutic wild type (wt) p53 can be rendered ineffective if it forms tetramers with mutated cancerous p53—the dominant negative (DN) effect [9]. Indeed, p53’s ability to induce apoptosis in aberrant cells was exploited in clinical trials in the late 1990s, but the therapy failed due to the DN effect, inability to overcome other mutated oncogenes, immunogenic issues with adenoviral delivery, and inefficient targeting of cancer cells [9].

**Fig. 2:**
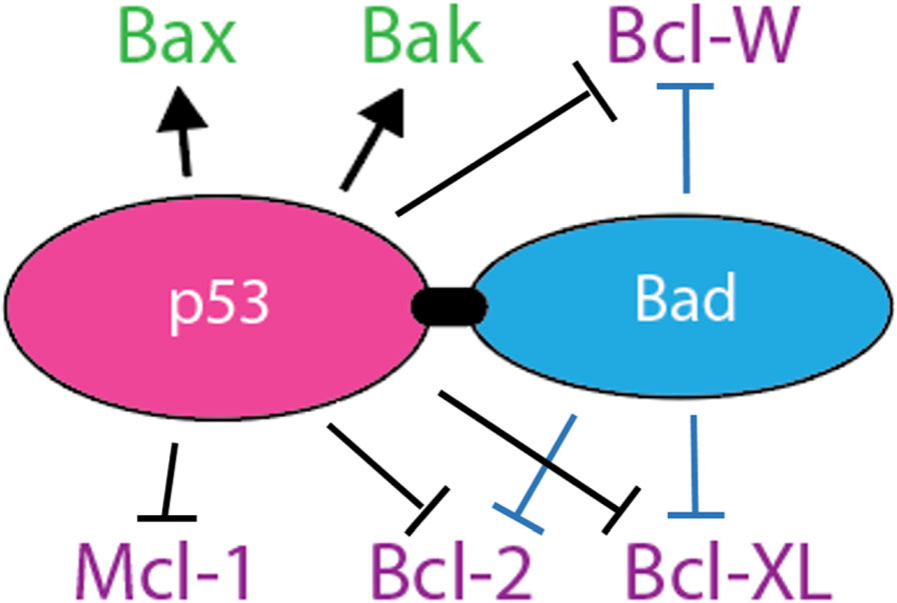
p53-Bad Fusion Construct. p53 both activates pro-apoptotic Bak and Bax as well as inactivates anti-apoptotic Bcl-W, Mcl-1, Bcl-2, and Bcl-xL [38]. Bad inactivates Bcl-W, Bcl-2, and Bcl-xL [39]. Thus, a fusion construct increases pro-apoptotic activity through dual inhibition of anti-apoptotic factors, as shown quantitatively previously by our lab (data unpublished, submitted for review)

To overcome p53 gene therapy failure, we propose a cancer-specific, mitochondrially-targeted p53 gene construct to treat ovarian cancer. Recent work in our lab has focused on fusing p53 to BH3 proteins, which both contain a mitochondrial targeting signal (MTS) [12] as well as potently enhance the apoptotic activity of p53. In particular, the apoptotic protein Bad—which can block anti-apoptotic proteins Bcl-xL, Bcl-2, and Bcl-W [13, 14]—has been tested in several ovarian cancer cell lines and shows greatly enhanced apoptotic activity compared to wt p53 when fused to p53 (Fig. 2) (unpublished data, submitted for review). Thus, not only can mitochondrially-targeted p53 overcome the DN effect, it also displays increased apoptotic induction over wt p53. Additionally, p53-Bad can be mutated (p53-Bad*) to replace two serine residues (112 and 136) with alanines, which increases mitochondrial localization by removing phosphorylation sites that can lead to sequestration of Bad in the cytoplasm [15]. Due to their multiple targets and dual nature, our novel p53-Bad hybrids should cause functional apoptosis in any cancer cell regardless of their p53 mutation status or the presence of other oncogenes.

**Fig. 3:**
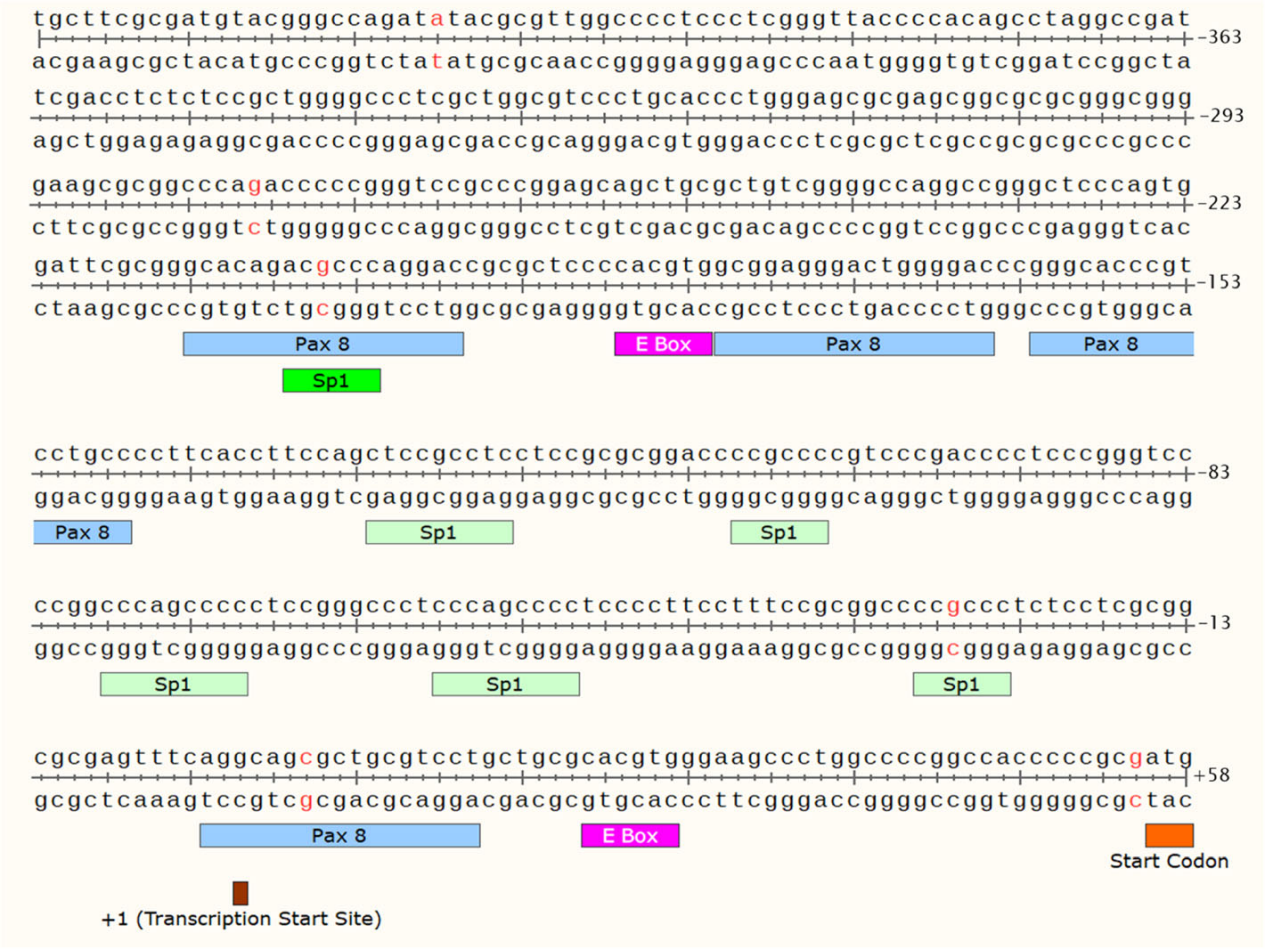
hTERT Promoter Region. Transcription factor binding sites for c-Myc (E-boxes), Pax 8, and Sp-1 are shown [18]. Base pairs highlighted in red indicate the start/stop points for the various hTERT lengths referenced in the text. Figure made with SnapGene software (from GSL Biotech; available at snapgene.com)

Though the p53-Bad hybrids show strong apoptosis in a variety of ovarian cancer cell lines, cancer specificity must be achieved in order to develop them into a feasible therapy. Unlike small molecule chemotherapy, gene therapy can employ cancer-specific promoters in order to control the expression of the therapeutic construct. The human telomerase reverse transcriptase (hTERT) promoter controls the expression of hTERT, a catalytic subunit of telomerase that is highly specific to many types of cancer cells [16], including ovarian cancers [17]. hTERT is regulated by many different transcription factors [16], with some of the most prominent including c-Myc, Pax, and Sp-1. c-Myc, along with its dimerization partner Max, binds to segments of DNA called E-boxes (CACGTG, see Fig. 3), where it recruits histone acetyltransferases to activate associated promoters [18]. c-Myc is upregulated in a large subset of ovarian cancers (up to 60% [19]) and is associated with decreased patient survival [19]. Pax8, a paired box developmental gene, is a transcriptional activator with several binding sites within the hTERT promoter (Fig. 3) and the Pax family is known to confer survival advantages on cancer cells when upregulated [20]. Sp-1 co-activates with c-Myc [16] but also activates hTERT through its own binding sites (Fig. 3).

Recently, the Ran (Ras-related nuclear protein) and Brms1 (breast cancer metastasis suppressor 1) promoters were shown to display high activity—comparable to the CMV promoter—as well as cancer specificity in vivo in ovarian cancer (Bg-1 cells), prostate cancer (PC-3 cells) and breast cancer (MCF-7 cells) [21]. Ran, a small GTPase, participates in nuclear transport and is a key player in mitosis that helps to form the mitotic spindle. The cancer-specificity of the Ran promoter is suspected to stem from Ran’s mitotic activity, as rapidly dividing tumor cells require greater amounts of mitotic factors [21]. Brms1 inhibits tumor cell migration by suppressing NF-κB [21]. Though the cancer-specific function of the Brms1 promoter may be somewhat surprising considering Brms1’s anti-tumorigenic function, if a cancer cell has not managed to mutate the Brms1 pathway then high Brms1 activity is logical [21].

Though the vast majority of HGSCs have p53 mutations [6–8], these mutations differ drastically between individual cases. Thus, testing cell lines with a variety of p53 mutation statuses is essential to ensure that p53-Bad/p53-Bad* fusion gene therapy will function regardless of p53 status. Skov3 cells contain a H179R mutation and do not express p53 mRNA, thus they are considered p53 null [22, 23]. Ovcar3 cells contain a R248Q mutation [22], which is a dominant negative mutation in the DNA binding domain of p53 [24]. Kuramochi cells, considered to have one of the most closely matching genetic profiles to primary patient tumors of all commercially available ovarian cancer cell lines [25], contain a dominant negative D281Y mutation that likely causes aggregation of p53 [22, 26]. BJ cells, normal human fibroblasts immortalized with telomerase, contain functional wild type p53 [27].

Other groups have tested a variety of lengths of the hTERT promoter in various types of cancer, and the Ran and Brms1 promoters have been shown to be successful in several different cell lines. This paper aims to test these past successes in a panel of ovarian cancer cell lines with varying p53 statuses. This should result in discovery of the best promoter to impart both cancer specificity as well as potent apoptosis, regardless of p53 status, in ovarian cancer cell lines in preparation for a therapeutic that will be effective in a wide range of patient tumors.

## Results

### hTERT promoters

**Fig. 4:**
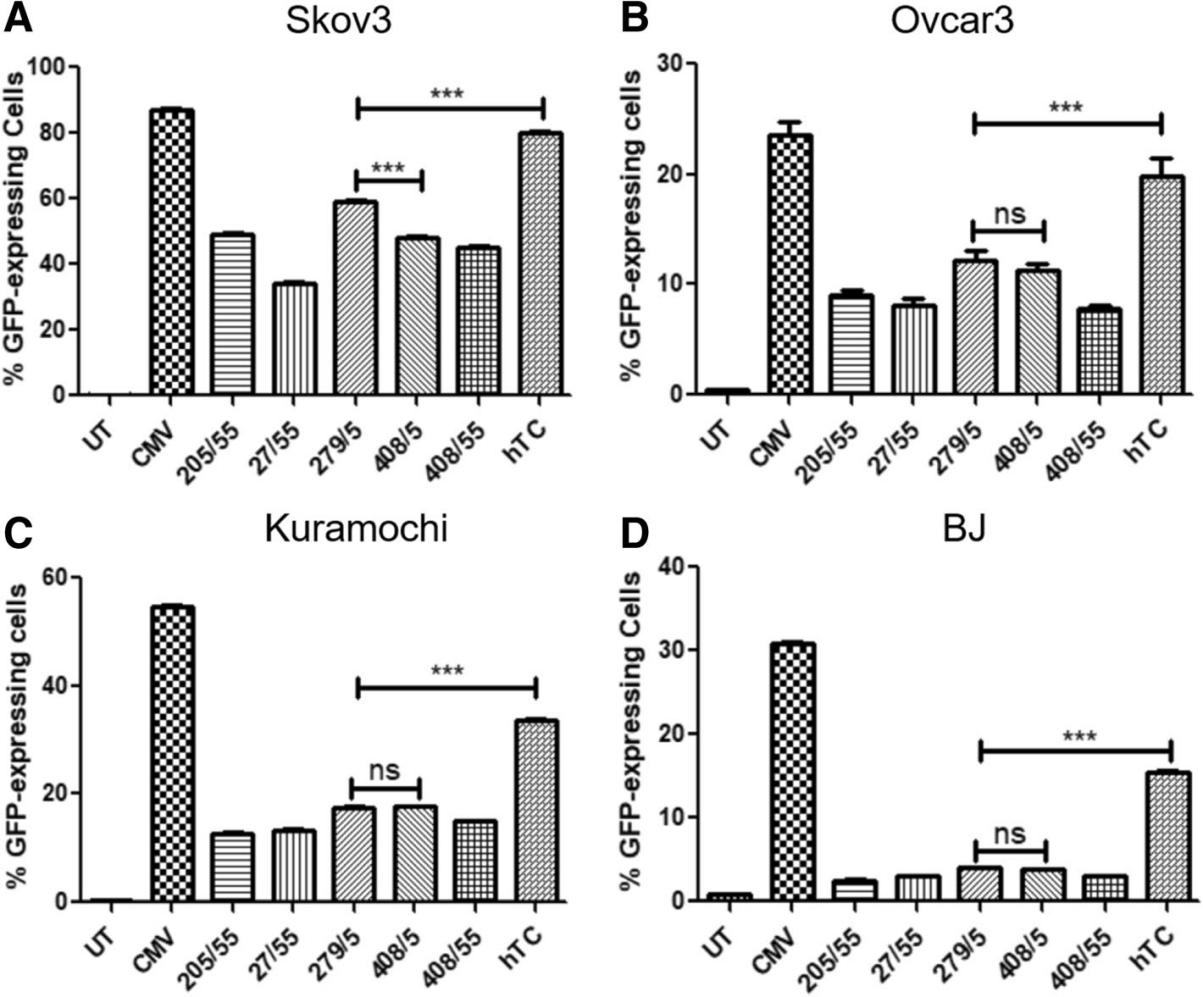
Expression of hTERT Promoters in Ovarian Cancer Cell Lines. **a**: In p53 null ovarian cancer cells (Skov3), expression of the hTC fusion promoter was significantly higher than all other hTERT constructs, with − 279/+ 5 yielding significantly higher expression than any of the other hTERT only constructs (*P* < 0.0001 for − 279/+ 5 versus all other constructs). **b**: In p53 dominant negative gain of function ovarian cancer cells (Ovcar3), hTC yields significantly higher expression than all other constructs, with − 279/+ 5 trending higher than the other hTERT constructs. **c**: In p53 dominant negative aggregation mutant ovarian cancer cells (Kuramochi), hTC yields significantly higher expression than all other constructs, with − 279/+ 5 expressing similarly to − 408/+ 5 and both trending higher than the other hTERT constructs. **d**: In normal human fibroblasts (BJ), hTC expression is significantly higher than all other hTERT constructs, and total expression levels of all hTERT constructs are lower than in ovarian cancer cell lines. For each graph, *n* = 3 and one-way ANOVA analysis yielded a *P* value of < 0.0001. Tukey post-tests were performed on all data sets, with *** indicating a *P* value of < 0.001

**Fig. 5:**
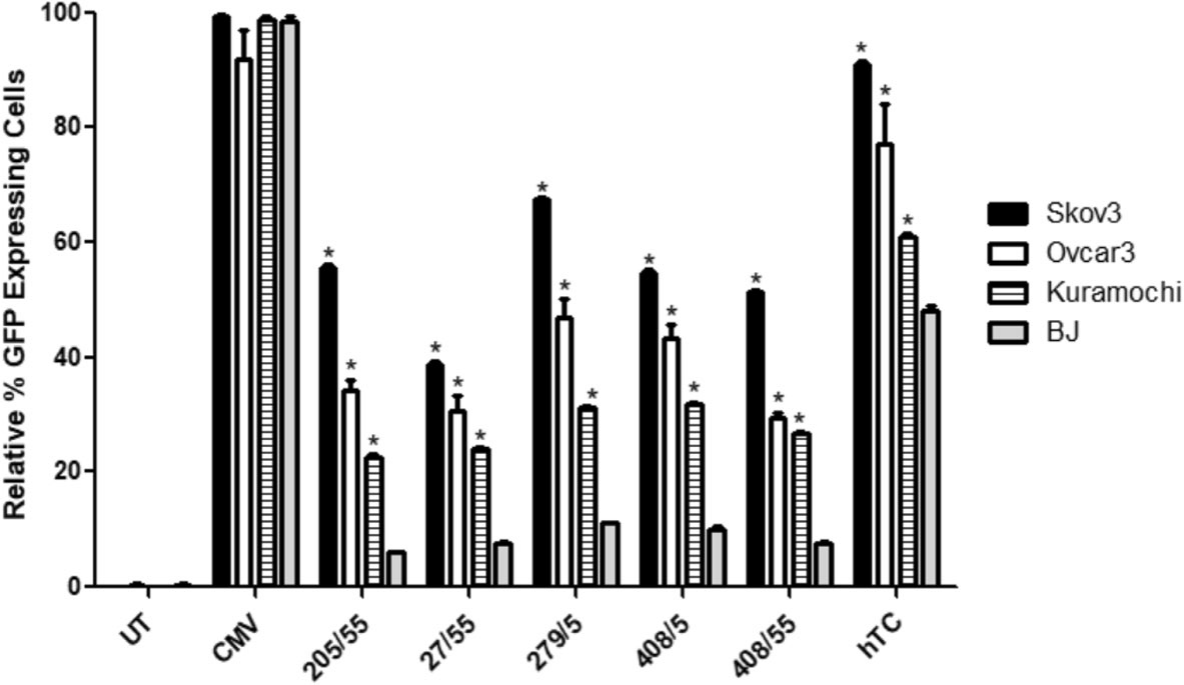
Cancer Specificity of hTERT Promoters. Every promoter indicates significantly lower expression in the normal cells versus every cancer cell line, while the untreated and CMV control columns indicate no significant different between normal (BJ) cells and any of the ovarian cancer cells. For each column *n* = 3, and statistical analysis was performed using 2-way ANOVA and Bonferroni’s post-test. Each * corresponds to a *P* value < 0.001 when the respective ovarian cancer cell column is compared to the normal cell (BJ) column in the same category with Bonferroni’s post-test

A variety of lengths of hTERT promoter (− 205/+ 55, − 27/+ 55 [28], − 279/+ 5 [29], − 408/+ 5 [29], and − 408/+ 55) as well as the hTC hTERT/CMV enhancer fusion promoter [30] were cloned into an EGFP-expressing plasmid, then tested in Skov3 (p53 null) [22], Ovcar3 (p53 dominant negative, gain of function R248Q mutant) [22], and Kuramochi (p53 dominant negative D281Y aggregation mutant) [22] ovarian cancer cells as well as BJ normal human fibroblasts (p53 wild type) [27]. 48 h post-transfection, all cell lines displayed the highest GFP expression levels under the non-specific CMV promoter (as expected), with the second highest expression of GFP powered by the hTC fusion promoter (Fig. 4a-d, 2^nd^ and last bars). The Skov3 cells displayed the highest hTERT-only GFP expression under the − 279/+ 55 hTERT promoter (Fig. 4a, 5^th^ bar), and all other cell lines displayed no significant difference between − 279/+ 5 and the next highest hTERT promoter section (Fig. 4b-d). Comparison of the cell lines by normalizing the untreated control to 0% and the CMV promoter to 100% (Fig. 5) showed significantly higher expression in all promoters across all cancer cell lines when compared with normal BJ cells. The hTERT-only promoters showed greater specificity than the hTC promoter; the BJ cells displayed about 75% expression compared to the next lowest—Kuramochi—cells under control of hTC but only about 30% expression compared to Kuramochi under control of each of the hTERT-only promoter sections (Fig. 5).

**Fig. 6:**
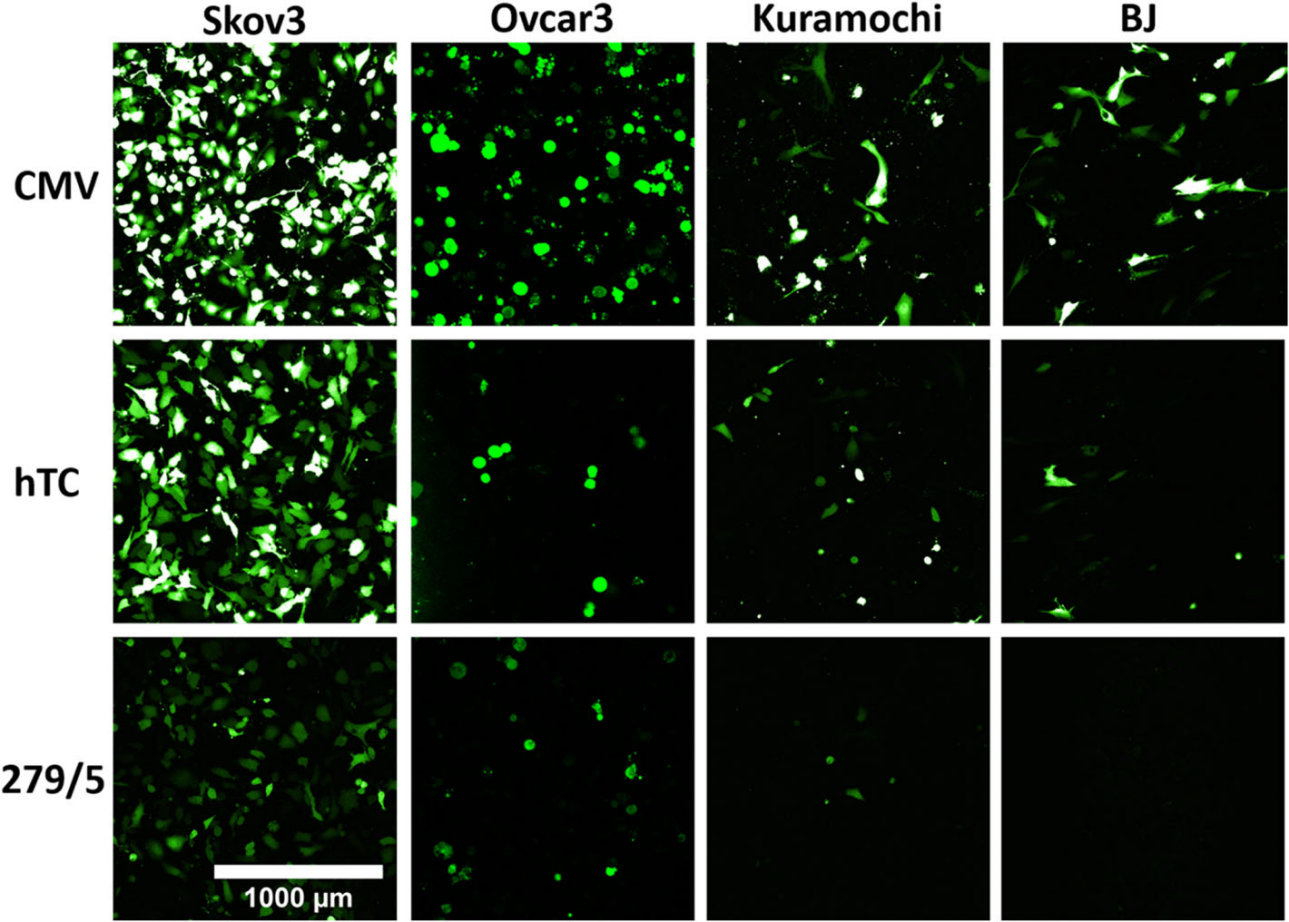
Fluorescent Images of Top 2 hTERT Promoters. All cell lines displayed a qualitative decrease in expression levels from CMV to hTC and hTC to − 279/+ 5. Data could not be quantified due to the difference in expression levels (brightness) between CMV and − 279/+ 5—either CMV would be overexposed, as shown, or − 279/+ 5 would be non-visible. Kuramochi and Ovcar3 are slightly under-represented in the photos, as their confluency was lower (~ 15% and ~ 30–50%, respectively. See text for more details). Scale bar based on Nikon A1R microscope settings

Microscopy of each cell line transfected with GFP under control of CMV (positive control), hTC and 279/5 (the 2 most promising promoter candidates) qualitatively shows the same expression trend as before—decreasing expression from CMV (Fig. 6, top panels) to hTC (Fig. 6, middle panels) to − 279/+ 5 (Fig. 6, bottom panels). Additionally, there is qualitative evidence of cancer specificity, with less expression of EGFP in BJ cells under the hTC promoter than in the cancer cell lines, and no EGFP expression detected under the − 279/+ 5 promoter in the BJ cells (Fig. 6, last column). It should be noted that the BJ and Skov3 cells had higher confluency (65 and 70%, respectively) compared with the Ovcar3 cells (− 279/+ 5 50% confluent, hTC and CMV 30% confluent) and Kuramochi cells (15% confluent).

### Ran/Brms1 promoters

**Fig. 7:**
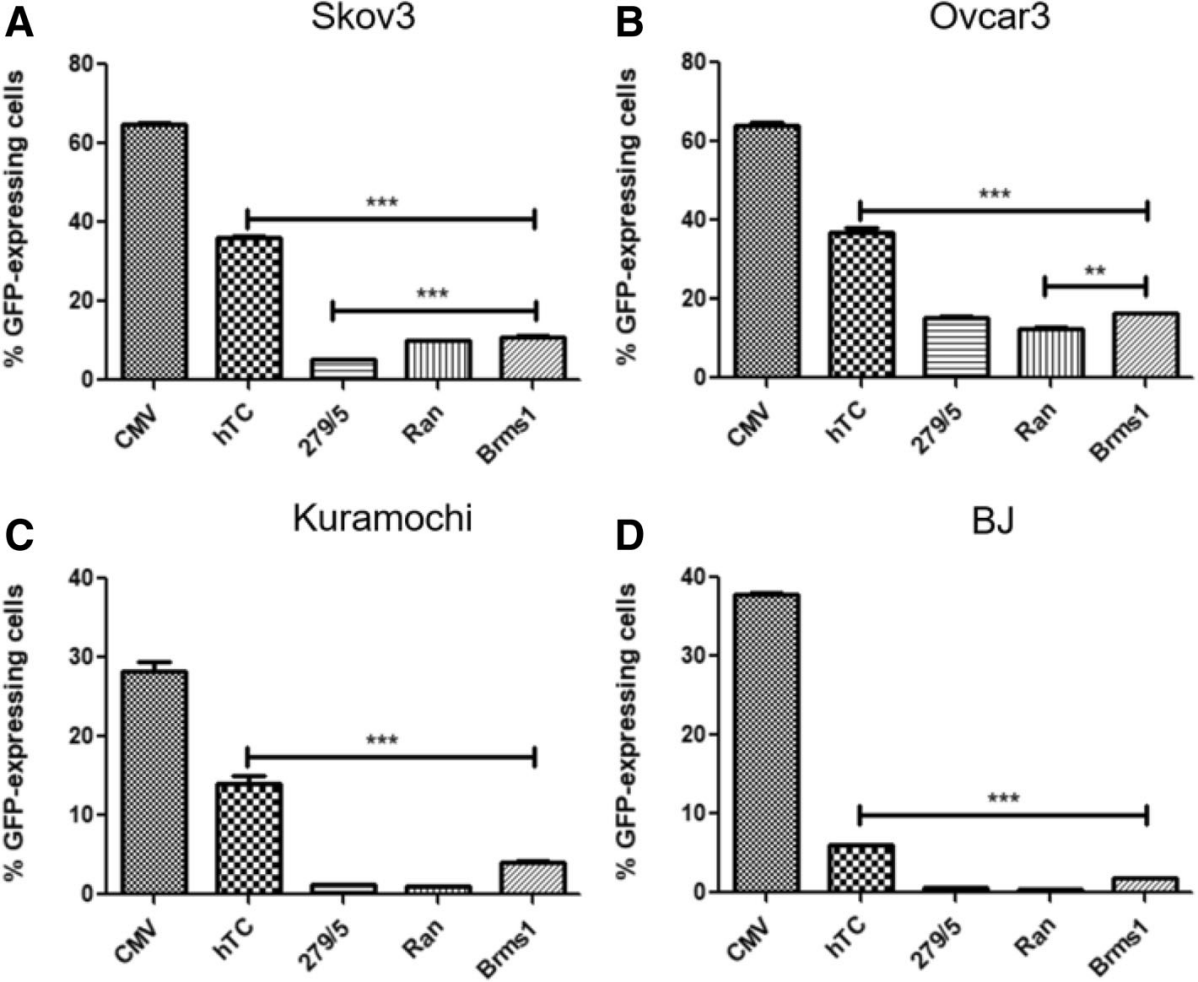
hTC, − 279/+ 5, Ran, and Brms1 Promoters. hTC displayed significantly higher expression levels than all other cancer-specific promoters in all four cell lines. Brms1 displayed the second highest expression levels in all cell lines but Ovcar3, where it showed very similar expression to the − 279/+ 5 promoter. Each column represents *n* = 3, and all data were analyzed with 1-way ANOVA and Tukey’s post-test. *** indicates *p* < 0.001, while ** indicates *p* < 0.01

**Fig. 8:**
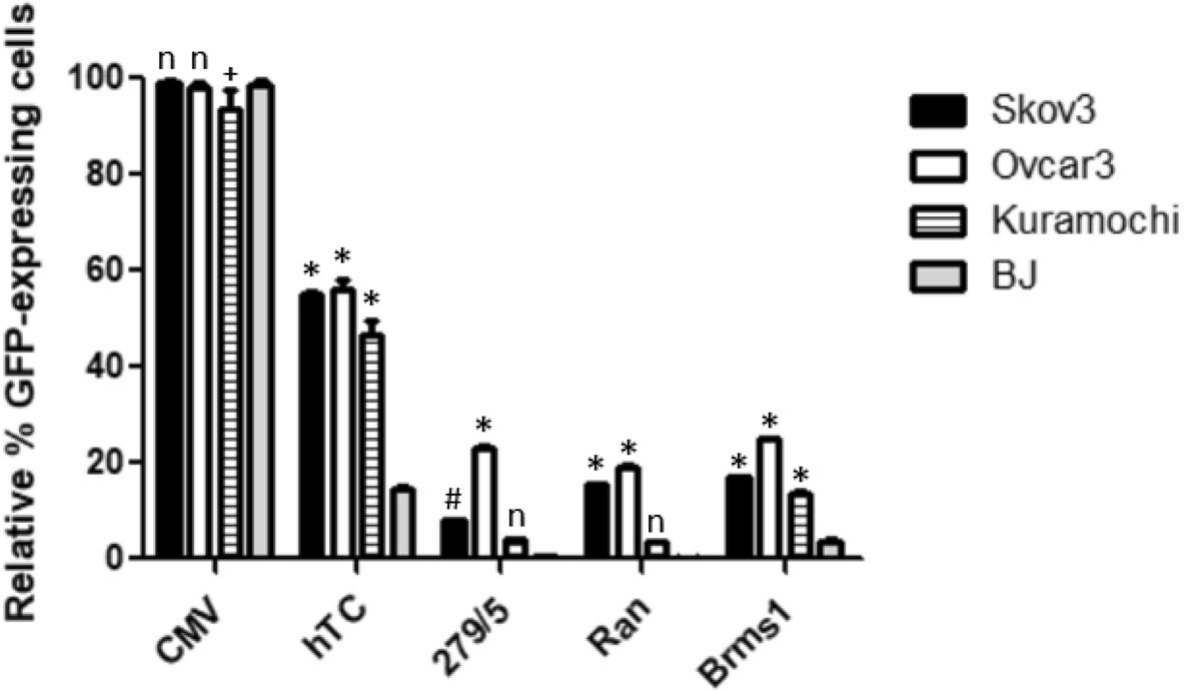
Cancer Specificity of hTC, − 279/+ 5, Ran, and Brms1 Promoters. hTC and Brms1 display robust cancer specificity in all three cancer cell lines compared with normal BJ cells. Ran and − 279/+ 5 demonstrate cancer specificity in Skov3 and Ovcar3 cell lines (the degree of specificity is low in Skov3 for − 279/+ 5), but not in Kuramochi cells compared with normal BJ cells. For each column *n* = 3, and statistical analysis was performed using 2-way ANOVA and Bonferroni’s post-test. Each * corresponds to a *P* value < 0.001, each # a *P* value of < 0.01, each + a *P* value of < 0.05, and each “n” to a non-significant *P* value when the respective ovarian cancer cell column is compared to the normal cell (BJ) column in the same category with Bonferroni’s post-test

Ran and Brms1, along with hTC and − 279/+ 5, were tested for GFP expression levels in the same four cell lines as before (Fig. 7), and once again hTC showed higher expression levels than any other cancer-specific promoter in all four cell lines (Fig. 7a-d, 2^nd^ bar). Overall, Brms1 fared second best (Fig. 7, last bar), with higher than or similar expression to − 279/+ 5 (Fig. 7, 3^rd^ bar) and Ran (Fig. 7, 4^th^ bar) in all four cell lines. The hTC (Fig. 8, 2^nd^ set of bars) and Brms1 (Fig. 8, last set of bars) promoters also displayed cancer specificity in the form of significantly higher GFP expression in all cancer cells when compared with normal BJ cells, while − 279/+ 5 and Ran showed specificity in Skov3 and Ovcar3 but not Kuramochi cells (Fig. 8).

### TMRE assays

**Fig. 9:**
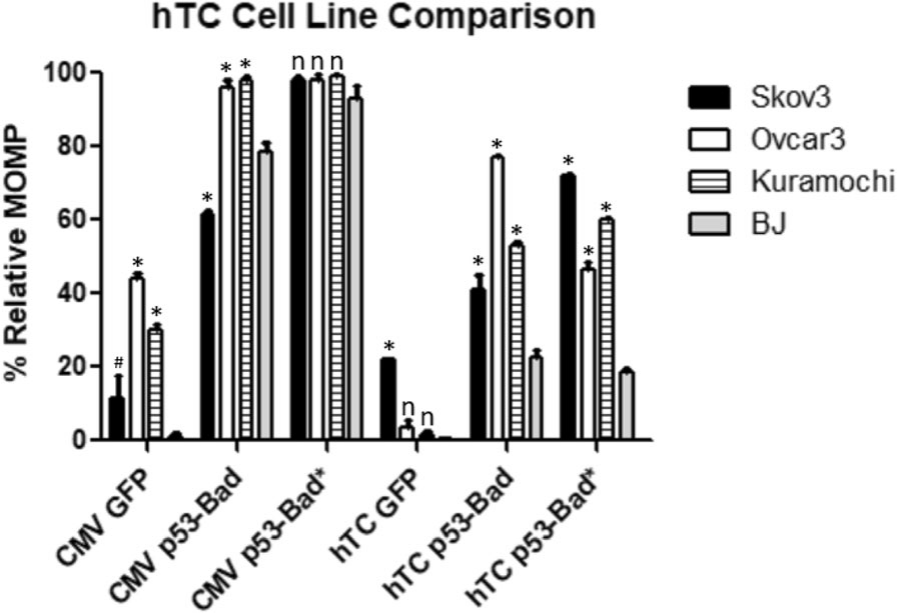
hTC TMRE Cell Line Comparison. p53-Bad* constructs under control of the CMV promoter show no significant difference between any of the cell lines, while all cancer cell lines show significantly higher apoptosis in hTC-controlled p53-Bad* compared with normal BJ cells. For each column *n* = 3, and the data were analyzed using 2-way ANOVA and Bonferroni’s post-test. Each * corresponds to a *P* value < 0.001, each # a *P* value of < 0.01, and each “n” to a non-significant *P* value when the respective ovarian cancer cell column is compared to the normal cell (BJ) column in the same category with Bonferroni’s post-test

**Fig. 10:**
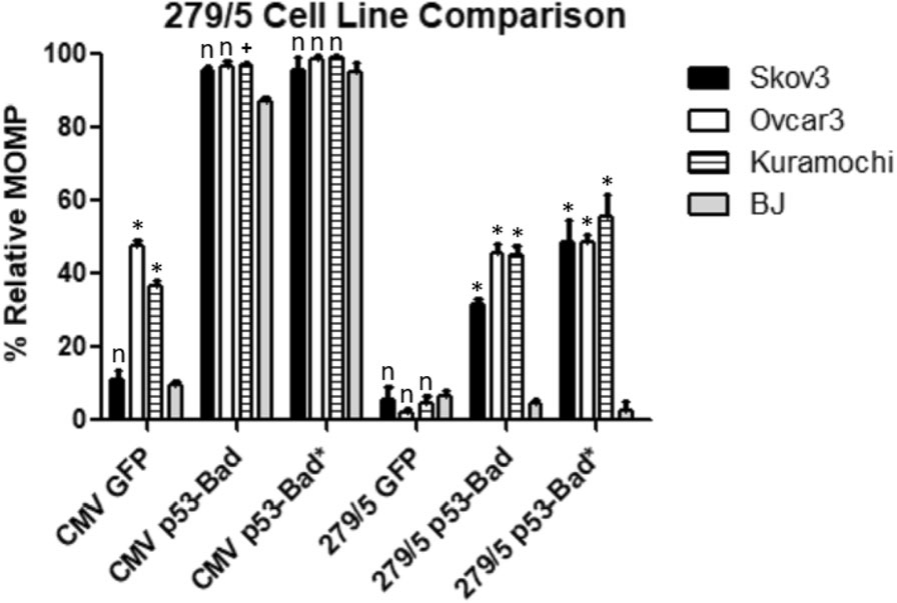
–279/+ 5 TMRE Cell Line Comparison. p53-Bad* constructs under control of Brms1 show significantly more apoptosis in all ovarian cancer cell lines than those under control of CMV compared to normal BJ cells. For each column *n* = 3, and the data were analyzed using 2-way ANOVA and Bonferroni’s post-test. Each * corresponds to a P value < 0.001, each + a *P* value of < 0.05, and each “n” to a non-significant *P* value when the respective ovarian cancer cell column is compared to the normal cell (BJ) column in the same category with Bonferroni’s post-test

**Fig. 11:**
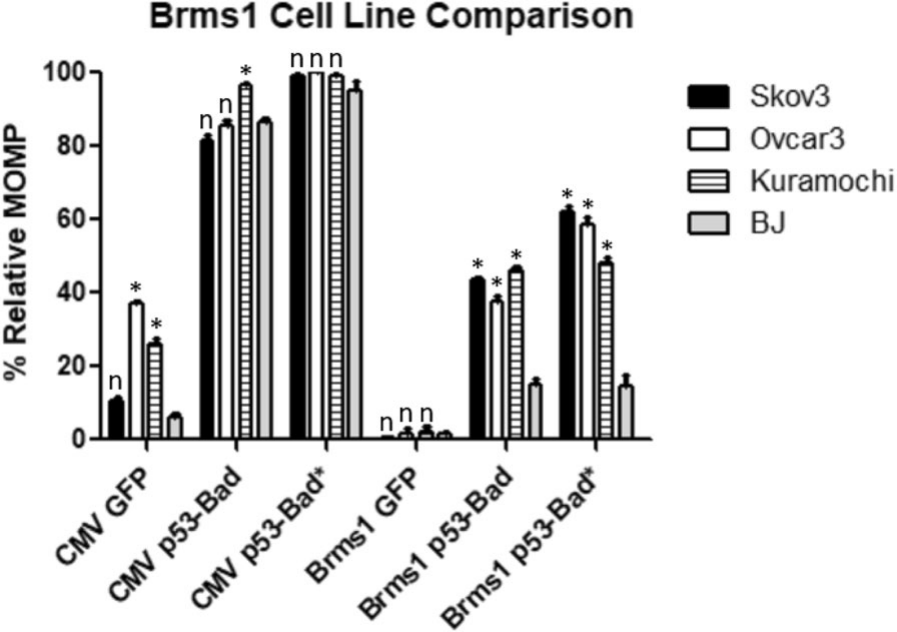
Brms1 TMRE Cell Line Comparison. p53-Bad* constructs under control of Brms1 show significantly more apoptosis in all ovarian cancer cell lines than those under control of CMV compared to normal BJ cells. For each column *n* = 3, and the data were analyzed using 2-way ANOVA and Bonferroni’s post-test. Each * corresponds to a *P* value < 0.001, and each “n” to a non-significant *P* value when the respective ovarian cancer cell column is compared to the normal cell (BJ) column in the same category with Bonferroni’s post-test

p53-Bad and p53-Bad* fusion constructs under the control of hTC, − 279/+ 5, Ran, or Brms1 were tested in all four cell lines for mitochondrial outer membrane permeabilization (MOMP), a hallmark of early intrinsic (mitochondrial) apoptosis (Figs. 9, 10 and 11). Cells were gated for GFP to determine whether or not they were expressing the construct, then for TMRE to determine mitochondrial apoptosis. The hTC, − 279/+ 5, and Brms1 promoters all display significantly higher levels of killing by p53-Bad* in cancer cells versus normal cells (Figs. 9, 10 and 11), while the CMV promoter consistently shows no significant difference between killing by p53-Bad* in cancer versus normal cells. p53-Bad shows significantly higher killing in all cancer cells under control of all 3 cancer-specific promoters, but displays some variation in killing under control of the CMV promoter (Figs. 9, 10 and 11). Transfection levels of Ran-driven constructs were too low in several cell lines for collection of a statistically significant number of cells (data not shown).

### Time point studies

**Fig. 12:**
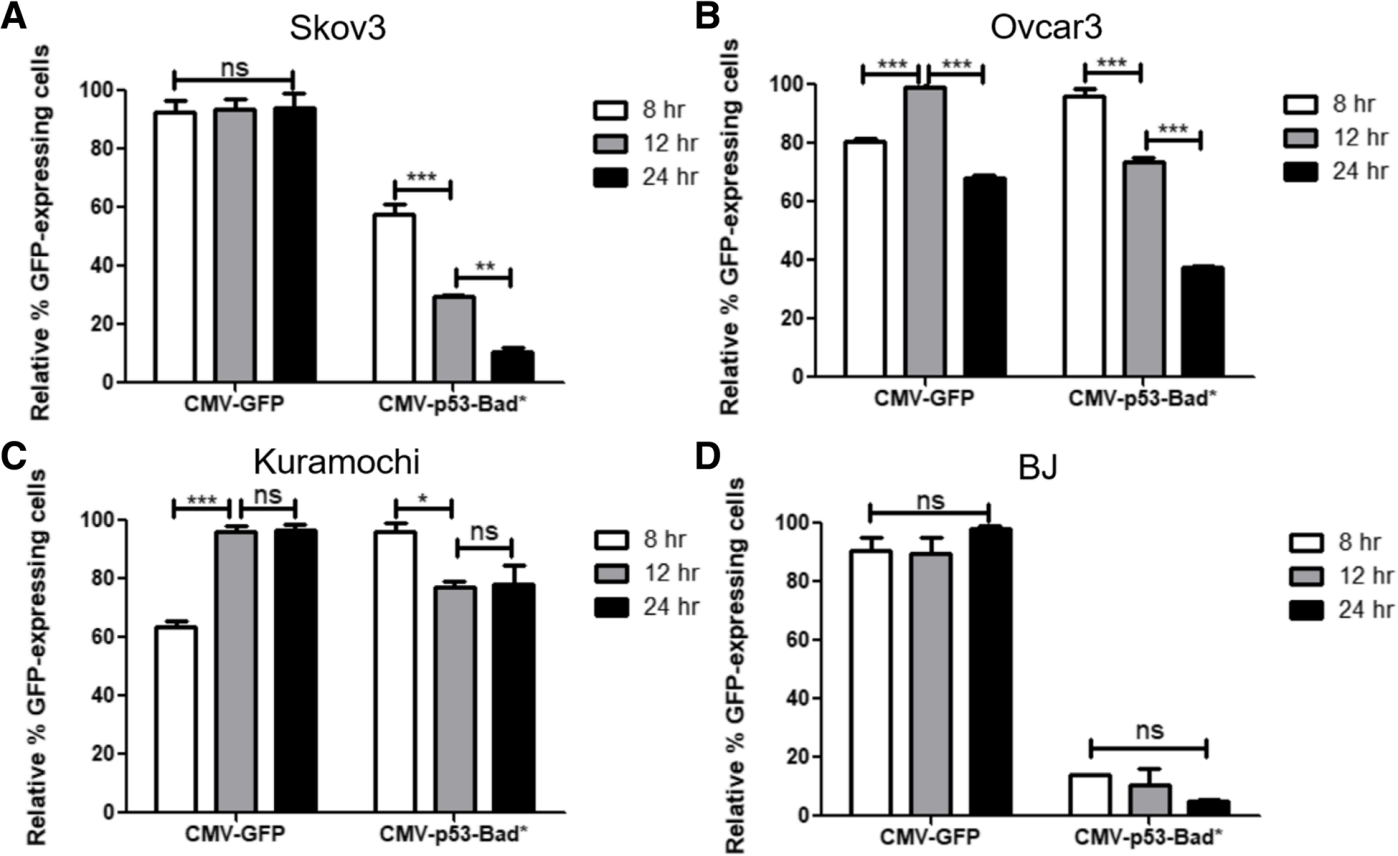
Expression Levels at 8, 12, and 24 h. In Skov3 cells (**a**), GFP expression under the CMV promoter remains constant at 8, 12, and 24 h time points while p53-Bad* expression under CMV decreases significantly at each time point. Ovcar3 cells (**b**) display maximum expression of GFP at 12 h and p53-Bad* at 8 h. Kuramochi cells (**c**) show maximum expression of GFP at 12 h with no significant difference at 24 h, while p53-Bad* expression maximizes at 8 h and drops significantly at later time points. BJ cells (**d**) show no significant differences in GFP or p53-Bad* expression levels when compared across time points, though the p53-Bad* expression trends downward at longer time points compared to earlier. For each column *n* = 3 (except the Kuramochi 24 h time point, where *n* = 6), and the data were analyzed using 2-way ANOVA and Bonferroni’s post-test. Each *** corresponds to a *P* value < 0.001, each * to a *P* value < 0.05, and each “ns” to a non-significant *P* value

At the 24-h time point used in the TMRE assays (Figs. 9, 10 and 11), all p53-Bad/p53-Bad* constructs displayed much lower expression levels than their GFP-only counterpoints, regardless of the promoter used. This made collection of data difficult, as more cells had to be collected in the p53-Bad/p53-Bad* samples, or, in the case of the Ran experiments, not enough GFP-expressing cells were present to successfully complete the experiment. Thus, CMV-GFP versus CMV-p53-Bad* expression was tested at 8, 12, and 24 h time points. In the Skov3 cells (Fig. 12a), CMV-GFP showed no significant difference in the number of cells expressing the construct across the time points, while CMV-p53-Bad* showed a significant decrease in the number of cells expressing the construct as time went on. Ovcar3 cells (Fig. 12b) showed peak CMV-GFP expression in cells at 12 h, while CMV-p53-Bad* cell expression peaked at 8 h and steadily decreased thereafter, with only around half of the number of cells expressing the construct at 24 h compared with the CMV-GFP construct. Kuramochi cells (Fig. 12c) showed increasing numbers of cells expressing CMV-GFP from 8 to 12 h, then steady expression from 12 to 24 h, while the number of cells expressing CMV-p53-Bad* peaked at 8 h, then dropped at 12 h and stayed steady up to 24 h. BJ cells (Fig. 12d) showed no significant difference in the number of cells expressing either construct over the different time points, though the number of cells expressing CMV-p53-Bad* was much lower than the number expressing CMV-GFP.

## Discussion

Unlike small molecule therapies, gene therapeutics have a built-in specificity mechanism—promoters—that, if properly harnessed, can lead to a new age of cancer therapies with little to no toxic side effects. The hTERT promoter is one of the most universal cancer-specific promoters and has previously been shown to be ovarian cancer specific [31], though different studies have reported different lengths of hTERT to be the most successful promoter [28, 29]. To this end, five different lengths of hTERT (− 205/+ 55, − 27/+ 55 [28], − 279/+ 5 [29], − 408/+ 5 [29], and − 408/+ 55) and one hTERT/CMV enhancer fusion promoter (hTC) [30] were cloned and tested in three ovarian cancer cell lines (Skov3, p53 null; Ovcar3, p53 R248Q DN; Kuramochi, p53 D281Y DN) and one normal cell line (BJ, p53 wt). Promoter − 27/+ 55 was chosen because it was the minimal domain of hTERT reported as effective [28], promoter − 205/+ 55 because it contains both E boxes and all Sp1 binding sites (Fig. 3), promoters − 279/+ 5 and − 408/+ 5 because they were reported by Horikawa et al. to have the highest expression of all their tested hTERT promoters [29], and promoter − 408/+ 55 was designed to encompass all the area covered by the other promoters. hTC was chosen because it was reported to achieve higher activity than hTERT-only promoters by combining the − 400/+ 54 length of hTERT with a CMV enhancer (− 1017/− 901) as detailed in the literature [30]. Because the gene therapy construct being developed by our lab is a p53-BH3 fusion construct, ovarian cancer cells were chosen for their varying p53 statuses: p53 null, p53 dominant negative (DN) mutant, and p53 DN/aggregation mutant for Skov3 [23], Ovcar3 [23], and Kuramochi [32], respectively. Additionally, Kuramochi cells are advantageous because they have the highest genetic similarity to primary HGSC tumors out of a large panel of ovarian cancer cell lines [25]. Due to the lack of a commercially available normal human ovary cell line, normal human fibroblasts (BJ cell line, p53 wt^27^) were used to measure promoter activity in normal cells.

All five hTERT lengths as well as the hTC promoter and CMV promoter (as a positive control) were cloned into plasmids expressing EGFP and transfected into all four cell lines. A separate plasmid containing CMV was used as a control because when a bicistronic plasmid was used, the CMV and cancer-specific promoters interfered with one another. 48 h post-transfection, the CMV promoter (as expected) displayed the highest expression level in all four cell lines, with most cell lines following the general pattern of hTC displaying the next highest expression, then − 279/+ 5 and − 408/+ 5, followed by the other three hTERT promoters (Fig. 4). Skov3 cells veered somewhat from this pattern, displaying much higher transfection levels in general and significantly higher − 279/+ 5 expression compared to that of − 408/+ 5, with − 205/+ 55 and − 408/+ 55 displaying comparable expression levels to − 408/+ 5 (Fig. 4a). Because − 205/+ 55 and − 408/+ 55 both contain two E-boxes (c-myc binding sites) rather than one, it is possible that variations in c-myc expression could be the reason behind this effect; Kuramochi [33, 34] and Ovcar3 [35] have both been reported to have normal c-myc expression, and Skov-3 has fairly normal c-myc expression but slightly higher than Ovcar3 [35].

When analyzed for cancer specificity compared with the normal BJ cell line, all of the hTERT promoters and the hTC promoter displayed significant cancer specificity compared to the CMV promoter (Fig. 5). The hTC promoter showed the highest expression levels in each ovarian cancer line but was not as cancer-specific as the hTERT-only promoters—normal BJ cells showed about 75% expression under hTC compared with Kuramochi (Fig. 5), but only about 30% expression under the hTERT-only promoters compared to Kuramochi (the closest cancer cell line to BJ in terms of expression level as well as the cell line most similar to primary patient ovarian tumors [25]). Thus, hTC shows the highest expression for a cancer-specific promoter, but the hTERT-only promoters show greater cancer-specificity. Based on these results, hTC and − 279/+ 5 (the highest expression amongst the hTERT-only promoter sections) were chosen to move on.

In addition to testing the well-known hTERT promoter, we also wanted to test newly reported ovarian cancer-specific promoters Ran and Brms1 [21]. After cloning Ran and Brms1 into an EGFP plasmid, they were tested compared to CMV, hTC, and − 279/+ 5. EGFP expression under each promoter was measured at 24 h (hence the slightly lower expression numbers compared with the 48-h hTERT data) in anticipation of the 24 h time point used for the TMRE early apoptosis assay. Once again, hTC showed vastly higher expression levels than the other cancer-specific promoters in every cell line (Fig. 7). Interestingly, the relative expression levels of − 279/+ 5, Ran, and Brms1 differed between the three ovarian cancer cell lines, with Brms1 showing clear superiority in Kuramochi cells (Fig. 7c), Ran and Brms1 significantly outstripping − 279/+ 5 in Skov3 cells (Fig. 7a), and a relatively close race between − 279/+ 5 and Brms1 in Ovcar3 cells (Fig. 7b). All four promoters showed cancer specificity compared to the normal BJ cells with the exception of − 279/+ 5 and Ran in Kuramochi cells (Fig. 8). Because − 279/+ 5 previously showed cancer specificity in Kuramochi cells at 48 h (Fig. 5), it was decided to move all four promoters forward to test whether they displayed cancer-specific killing when used to drive expression of the p53-Bad gene constructs.

hTC, − 279/+ 5, Ran, and Brms1 were all cloned into plasmids expressing p53-Bad and p53-Bad*, constructs recently shown by our lab to cause potent apoptosis in ovarian cancer cell lines (data unpublished, submission under review). 24 h post-transfection, cells were assayed for mitochondrial outer membrane permeabilization, an indicator of early mitochondrial apoptosis. Cells were gated for proper morphology and then for EGFP, as p53-Bad and p53-Bad* both have a GFP protein fused to their N-terminus for detection. GFP-positive cells with TMRE are considered to be in the early stages of mitochondrial apoptosis. The − 279/+ 5 promoter displayed significant cancer-specific killing across all cell lines compared to CMV for both p53-Bad and p53-Bad*, though p53-Bad* showed higher killing overall—particularly in Skov3 cells—as well as a greater difference between cancer cells and normal cells (Fig. 10). This shows the potential to use − 279/+ 5 in combination with p53-Bad* for future treatments, as such a high degree of specificity would allow for much higher doses of therapeutic without toxic side effects for patients. The hTC and Brms1 promoters show fairly similar results; both show cancer-specific killing for both p53-Bad and p53-Bad* in all three cancer cell lines compared with normal cells, though the degree of specificity is not as high as in − 279/+ 5 (Figs. 9 and 11). This may be counter-balanced by the higher expression levels seen from Brms1 and hTC driven constructs, however—in practice it may be more practical to use a therapeutic that is slightly less specific but more likely to be expressed in more cancer cells. Additionally, p53-Bad* caused similar or more apoptosis compared with p53-Bad when controlled by cancer-specific promoters, with the exception of hTC in Ovcar3 cells, which showed much higher killing with p53-Bad than p53-Bad* (Fig. 9). Overall, these results indicate that − 279/+ 5, Brms1, and hTC as promoters expressing p53-Bad* should all be moved forward into in vivo studies in order to find the optimum balance of specificity and potency, while Ran—which had too low of expression levels for accurate measurement of MOMP—can be eliminated as a possible promoter. Each of the three promoters may be best suited to different functions, with − 279/+ 5 being ideal for applications where very high specificity is desired, such as a gene therapy with particularly toxic side effects. hTC, which had the highest overall transfection levels, may be ideal for a drug with lower toxicity, and Brms1 may represent a balance between the two.

Transfection levels of p53-Bad/p53-Bad* constructs were lower than EGFP, and cancer-specific promoters throughout this study displayed lower numbers of cells expressing the construct of interest compared to the CMV promoter. The lower expression levels from cancer-specific promoters can easily be explained by the relative strengths of the promoters, but the difference in EGFP versus p53-Bad/p53-Bad* expression was explored further through a series of time point studies at 8, 12, and 24 h. CMV-GFP expression stayed steady across all time points for Skov3 (Fig. 12a) and BJ (Fig. 12d), peaked at 12 h for Ovcar3 (Fig. 12b), and peaked at 12 h/stayed steady at 24 h for Kuramochi (Fig. 12c). In distinct contrast, CMV-p53-Bad* expression peaked at 8 h for all 4 cell lines (results were not significant in BJ cells), dropping to much lower levels by 24 h (Fig. 12a-d). This indicates that p53-Bad* inherently either expresses earlier than GFP or simply kills quickly enough for many transfected cells to no longer be detectable at later time points. This makes apoptosis difficult to measure, but shows promise for future in vivo experiments, where overall tumor death can be measured. Interestingly, the overall number of BJ cells expressing p53-Bad* are much lower relative to those expressing GFP than in the other cell lines, indicating that there may be some amount of natural cancer specificity inherent in the p53-Bad* construct as well as in the cancer-specific promoters, making moving this construct forward into in vivo studies even more promising.

## Conclusions

Overall, 8 cancer-specific promoters were tested in 4 different cell lines, with − 279/+ 5, hTC, and Brms1 being recommended for further use driving the p53-Bad* construct in in vivo ovarian cancer studies. -279/+ 5 is the most cancer-specific, but may be limited by low expression levels. hTC and Brms1 show slightly less cancer specificity but higher expression levels, with hTC showing the highest expression levels. Future experiments will explore the efficacy of these promoters with p53-Bad* in vivo to determine the best balance between expression levels and cancer specificity for this gene therapy construct, but all three promoters show promise as ovarian cancer-specific gene therapy drivers. -279/+ 5 may be ideal for highly toxic gene therapies, as it has particularly high cancer specificity, while hTC and Brms1 may work best for gene constructs that require less lowering of toxic side effects for normal cells—their higher expression levels would increase the reach of the gene constructs they drive. Additionally, in this new age of personalized medicine, having several promoters available means that genetic testing of a patient’s tumor could lead to choosing the promoter that best targets their particular disease; having several options ready to go means more patients have a greater chance of survival. Eventually, this area of research could lead to drastically lowered toxic side effects for patients, making it possible to increase therapeutic doses to levels that will better destroy cancer cells and thus improve overall survival for countless patients.

## Materials and methods

### Cloning

Various hTERT promoter lengths were cloned into an EGFP plasmid with self-designed primer sets using the transcription initiation site reported in Horikawa et al.^29^ and the pAdv/TERT vector (AddGene) as a template. Primer sets are as follows. -205/+ 55: TAGTTATTAATCCCAGGACCGCGCTCCCCAC (forward) and TTATATGCTAGCGGATCGCGGGGGTGGCCG (reverse) -27/+ 55: TAGTTATTAATGCCCTCTCCTCGCGGCGCGAG (forward) and GGTAGCGCTAGCGGATCGCGGGGGTGGCCGGGGC -279/+ 5: TAGTTATTAATATACGCGTTGGCCCCTC (forward) and GGTAGCGCTAGCGGATGCTGCCTGAAACTCGCG (reverse) -408/+ 5: TAGTTATTAATGACCCCCGGGTCCG (forward) and GGTAGCGCTAGCGGATGCTGCCTGAAACTCGCG (reverse) -408/+ 55: CTAAGCTATTAATCGATACGCGTTGGCCCCTC (forward) and GTAATGAGCTAGCTAGGCGGGGGTGGCCGGG (reverse). For hTC [30], a Geneblock (Genewiz) containing hTERT − 400/+ 54 and CMV − 1017/− 901 was ordered with AseI and AgeI cut sites on the ends, then digested and cloned into the EGFP plasmid. -279/+ 5 and hTC were cloned into a plasmid containing p53-Bad* using primer set TAGTTATTAATGACCCCCGG (forward)/ TACATACCGGTGCTGCCTGAAAC (reverse) for − 279/+ 5 and the digested Geneblock from before for hTC.

Brms1 and Ran were cloned using nested PCR [21]. Brms1 PCR was performed using primers CACGACGGAGATTCCCTGAG and CCGCATGCCCATGAACAAAA for the first round, and primers GTGTGTATTAATGCTAGCTCCCTCCCCTAATCTGAGAA and ATATATACCGGTACGGAGATTCCCTGAGA for the second round using the first round product as template. First round Ran PCR used primers ATTTGCGTCACTGGGGTTCC and GAGCGGAGGATGAAACGGGG, while the second round primers were GTGTGTATTAATGCTAGCACGCGTCCAGACTGCAAACA and ATATATACCGGTCGCGATACCTTCCAGAA. Genomic DNA extracted from BJ (normal human fibroblast) cells with the DNeasy Blood and Tissue Kit (Qiagen) was used as a template for both Brms1 and Ran.

### Cell maintenance, seeding and transfection

Skov3 human ovarian adenocarcinoma (a kind gift from Dr. Shawn Owen, University of Utah), Kuramochi human ovarian carcinoma (JCRB Cell Bank, Japan), Ovcar3 human ovarian adenocarcinoma (ATCC, Manassas, VA) and BJ normal human fibroblasts (ATCC, Manassas, VA) were all grown in flasks in monolayers and maintained with DMEM (Skov3) or RPMI (Ovcar3, Kuramochi, and BJ) media supplemented with FBS (10% for Skov3/Kuramochi, 20% for Ovcar3/BJ), 1% l-glutamine (Corning), and 1% penicillin-streptomycin (Gibco). Additionally, Ovcar3 cells were supplemented with 0.01 mg/ml bovine insulin (Sigma Aldrich). Cells were seeded in CellBIND 6-well plates (Sigma Aldrich) for assays or 2-well viewing chambers (Thermo Fisher Scientific) for microscopy at varying concentrations in order to account for the difference in growth rates between cell lines. The approximate number of cells seeded per well are as follows: 200,000 cells/well for Skov3, 300,000 cells/well for Ovcar3 and Kuramochi, and 500,000 cells/well for BJ. 24 h after seeding, cells were transfected with 1 pmol of DNA per well using the JetPrime transfection reagent (PolyPlus Transfection) according to manufacturer instructions.

### Microscopy

48 h after transfection, cell media was removed and replaced with PBS. Images were taken using NIS software on a Nikon A1R or Olympus FV1000 fluorescence confocal microscope with a 40x objective with a basic GFP filter.

### GFP expression assay

24 (Top four promoter experiments) or 48 (hTERT promoter experiments) hours after transfection, cell media was collected and cells were washed with PBS, then harvested with 0.25% Trypsin EDTA (ThermoFisher Scientific). Cells were then pelleted by centrifugation at 1000 RPM for 5 min, after which media was aspirated and cells were re-suspended in 300 μl PBS. The percentage of cells positive for GFP were measured on the FACSCanto-II (BD-Biosciences, University of Utah Flow Cytometry Core), where they were gated for proper morphology as well as GFP (excitation 488 nm, emission filter 530/35) and analyzed with FACSDiva software.

### TMRE assay

As done previously [36, 37], cell media was collected 24 h after transfection and cells were washed with PBS then harvested with 0.25% Trypsin EDTA (ThermoFisher Scientific). Cells were then pelleted by centrifugation at 1000 RPM for 5 min, after which media was aspirated and cells were re-suspended in 300 μl of 100 nM tetramethylrhodamine ethyl ester (TMRE, Invitrogen) in 1x Annexin-V buffer (Invitrogen). Cells were incubated at 37 degrees C for 30–45 min, then analyzed on the FACSCanto-II (BD-Biosciences, University of Utah Flow Cytometry Core), where they were gated for proper morphology, GFP (excitation 488 nm, emission filter 530/35), and TMRE (excitation 561, emission filter 585/15) with FACSDiva software.

### Statistical analysis

All statistical analysis was performed using GraphPad Prism version 5.01 for Windows, GraphPad Software, La Jolla California USA, http://www.graphpad.com. For Figs. 4 and 7, one-way ANOVA followed by Tukey’s post-test were performed on the data. For Figs. 5, 8, 9, 10, 11, and 12, two-way ANOVA followed by Bonferroni’s post-test were performed on the data.
